# Developing global health technology standards: what can other industries teach us?

**DOI:** 10.1186/1744-8603-9-49

**Published:** 2013-10-17

**Authors:** Hassan Masum, Rebecca Lackman, Karen Bartleson

**Affiliations:** 1 Sandra Rotman Centre, University Health Network and University of Toronto, 101 College Street Suite 406, Toronto, ON M5G 1L7, Canada; 2 Grand Challenges Canada, 101 College Street Suite 406, Toronto, ON M5G 1L7, Canada; 3 Synopsys, Inc, 700 East Middlefield Road, Mountain View, CA 94043, USA

**Keywords:** Global health, Standards, Interoperability, Affordability, Interface, Modularity, Diagnostics

## Abstract

**Background:**

There is a lack of effective and affordable technologies to address health needs in the developing world. One way to address problems of innovation and affordability is to design global health technologies to follow agreed-upon standards. This Debate article argues that we can better develop standards for global health technologies if we learn lessons from other industries.

**Discussion:**

The article’s Background section begins by explaining why standards are needed in global health. For example, if global health technologies can be modularized into independent interfacing parts, these parts can then interact via well-defined standards in a “plug and play” fashion. This can avoid development of mutually incompatible solutions by different organizations, speed the pace of innovation, unlock health systems from single providers and approaches, and lower barriers to entry. The Background then gives a brief primer on standards and discusses incentives for health standards. The article’s Discussion section begins with brief relevant cases of standards development from other industries, including electricity, container shipping, CD standards, Universal Serial Bus (USB), and the Internet. It then explores lessons from these and other industries that suggest how to develop standards for global health technologies. The remainder of the Discussion considers intellectual property and regulatory issues and standards-based global health business models, and ends with a checklist of considerations for health standards development leaders. (The associated Additional file discusses observations from standards development for cell phones and semiconductors, as well as challenges in the standards development process itself.) Throughout the article, point-of-care diagnostics are used as an illustrative example. An initiative is already underway to explore standardized diagnostics platforms.

**Summary:**

This Debate article aims to convince the reader that standards can benefit global health technologies if we learn lessons from other industries. The article draws from historical examples and the authors’ experiences to suggest principles, challenges, and opportunities in developing these standards. If implemented well, standardized platforms can lower barriers to entry, improve affordability, and create a vibrant ecosystem of innovative new global health technologies.

## Background

### The potential of standards for global health technologies

The general problem that motivates this article is the lack of affordable and effective health technologies to address key health needs in the developing world. A wide range of technologies are either available or under development, including medical devices, diagnostics, drugs, telemedicine units, prosthetics, mobility aids, health record and information systems, simulators, maternal and family health technologies, bednets, and disinfection systems [1-3]. While there are many examples of such technologies that have had positive impact on global health, there is still a strong need for technologies which are more accessible, socio-culturally appropriate, and effective [4,5].

Developing global health technologies has unique challenges [6]. Much of the funding for development and purchase of these technologies comes from foundations and governments, which face immense needs with constrained resources. The landscape is fragmented, with many entities creating new technologies. There is a need for robustness (to function in high-stress environments with respect to power, heat, dust, vibration, etc.) and simplicity (to be effectively used with minimal training, support, and repair facilities) [7].

The barriers to development and adoption of needed new technologies for global health may be classified into four general categories: scientific (e.g. research capacity), economic (e.g. low purchasing power), policy (e.g. regulatory regimes), and socio-cultural (e.g. issues around gender or religion) [8]. (We will see later how barriers from all four categories can hinder the development of global health technology standards.)

This article first briefly explains in the Background why global health technologies should be designed to follow agreed-upon industry-wide interface standards, and in so doing better address problems of innovation and affordability. The Discussion then argues that we can better develop standards for global health technologies if we learn lessons from other industries.

(By “global health”, we mean health objectives that transcend national boundaries and aim to improve the health of people worldwide in an equitable way. Since most of the population and disease burden of the world is in lower and middle income countries, global health is primarily concerned with health issues that are common in those countries [9].)

As we will see, health technologies can benefit from standardization for several reasons. If a health technology can be “modularized” into independent interfacing parts, each of these interfacing parts can be improved or substituted separately. All parts can then interact according to well-defined interoperability standards. Along with physical interoperability, standards may also address “semantic interoperability” – the correct and unambiguous transfer of relevant meaning as data passes between systems. More generally, standards provide common principles and norms which can make technology implementation more efficient and effective.

We will use point-of-care diagnostics for use in developing countries as an illustrative example throughout this article [10-13]. (Standardized diagnostics platforms for global health could lower costs, speed innovation, and save lives. An initiative with funding from the Bill and Melinda Gates Foundation and Grand Challenges Canada is underway [14], with over US$30 M committed.) Medical devices are another example of interest.

There is a vast literature on the importance of standards for public policy [15] and for economic and technological progress [16-18]. For instance, a detailed German study suggested that standards have a positive effect on trade, enhance international competitiveness, and provide societal benefits beyond those to individual companies [19]. A more recent estimate illustrated the importance of standards for economic growth in four European countries and twelve sectors [20], and two other studies found positive impacts on labor productivity in Britain [21] and Canada [22].

Open standards do not lock users in to a particular vendor. They form the basis of the Internet’s innovations, and have been advocated for many applications such as a smart energy grid, healthcare information systems, and public and government data [23,24]. We argue that they can be especially beneficial for health technologies for the developing world. They can avoid vendor lock-in, support new modular solutions which interoperate with an installed base of technologies and devices, and open the door for lower-cost providers (some based in developing countries themselves) to take part in health technology innovation without needing to create and manufacture an entirely new platform.

### A brief primer on standards

The British Standards Institution provides this definition of technological standards: “Put at its simplest, a standard is an agreed, repeatable way of doing something. It is a published document that contains a technical specification or other precise criteria designed to be used consistently as a rule, guideline, or definition [25].”

Two basic functions of standards are *interface* (or compatibility) and *quality* (or safety). In general terms, a standard’s interface specifications define how products interoperate with each other or with the rest of the world. In contrast, a standard’s quality specifications define product performance against quality or safety metrics. Many standards are primarily concerned with one or the other, and in this case we may speak of an “interface standard” (or “technical standard”) in contrast to a “quality standard”.

For health technologies such as drugs and medical devices, regulatory bodies have to date been less concerned with interface standards than with quality standards – for example, safety, efficacy, and the issue of counterfeit drugs [26]. Where not otherwise indicated, this article is focused on interface standards. A familiar example of an interface standard is Universal Serial Bus (USB), which reduced costs and complexity for both consumers and manufacturers and is discussed in more detail later in this article.

New health technologies normally require quality standards as soon as they reach users and patients. In contrast, for interface standards premature standardization and modularity may “lock in” to a suboptimal architecture or interface, which is difficult to displace [27]. When there is no clear understanding of what functionality is best, a premature standard can lock technology developers into an inferior approach and stifle innovation, or (if the standard is voluntary) simply become a white elephant that is never adopted. As such, a novel health technology that has not been field-tested is likely not suitable for standardization.

Upgrade paths can be designed in from the start for standards. Designing health technologies for backward compatibility requires consideration of which “interface elements” can stay the same across technology generations. This allows the internals to change while the interfaces do not. For example, a diagnostic platform designer might change diagnostic algorithms rapidly, while wanting to maintain physical “plug-compatibility” across platform generations.

Maturity and backward-compatibility are two criteria for a good standard. Other criteria include forward-compatibility, modularity, maintainability, device-independency, internationality, extensibility, and simplicity [28].

### Incentives for global health standards

One might argue that technology developers have often been motivated to *reduce* compatibility, in order to maintain competitive advantage and lock users into proprietary offerings. Are there really sufficient counterbalancing incentives to create standards for global health technologies?

Fortunately, the answer is yes: there are several incentives to create global health standards. First, *appreciation of the social value of interoperability*. Can a convincing case be made that interoperability and standards will lead to less expensive and more innovative products for end users? To more lives saved? An example might be the expansion of treatment possible if a standardized platform increases affordability.

Such an argument could attract product developers who value social returns for their own sake, and for the sake of the positive publicity and employee motivation they bring. Substantial public goodwill is enjoyed by those developing solutions for the poorest.

Second, *requirements from buyers.* A large buyer can tip the balance toward one particular standard, or toward standards in general. Global health technologies are often funded by a few large buyers such as health ministries or donors. Buyers might prefer standards in order to avoid lock-in, or to encourage innovation and cost reductions. Considerations for buyers include mustering sufficient buying power, avoiding preference for a premature or deficient standard, maintaining good relations with technology developers, and addressing regulatory considerations.

Third, *requirements from technology development funders*. Preference for interoperability and standards conformance might become part of the global health funding toolkit, complementing march-in rights and global access provisions [29].

It may be difficult to decide whether to exempt a non-conforming innovative approach from standards requirements, such as a cheap and robust paper diagnostic which cannot readily conform to plug or interface specifications. Standards should be written with such eventualities in mind. One tactic is to modularize the requirements themselves, so that one part can remain valid even if another becomes superseded by a new technological approach (akin to the idea of "severability" in law).

Fourth, *lower costs of innovation* leading to new technological and business approaches. Smaller entities can create a standards-conforming diagnostic, and license it to larger entities for scaled-up manufacturing and delivery. Larger entities can create a standard based on joint technology, allowing them to spread technology or create a new market faster.

A lowered cost of innovation enables more diverse technology creators, including those who may accept lower profit margins in exchange for greater product dissemination and impact. Ultimately, effective standards can even cause an entire market to grow.

Fifth, *open business models*. An example is the “open source” idea which originated in the software world. This denotes software whose source code is free to examine, use, or extend, under specified conditions. Open source has achieved well-known successes like Linux and Firefox in the software world. Its applications in health technologies to date are fewer, though there is significant potential [30,31].

Open standards facilitate collaborative approaches, which can speed innovation – for diagnostics, perhaps encouraging collaborative development of common algorithms and user interfaces. An open standard may be more readily adopted if core implementation pieces are made available in an open source way [32]. If leading organizations are willing to open up their technology while giving up some control, and a “coalition of the willing” can be instigated, then open business models can help develop standards and solutions.

Sixth, *social and cultural factors*. As an example, the adoption of open source software has been boosted in some countries by the desire to adapt software to local needs, become less dependent on imported solutions, and enable local innovators. Similar incentives may help “make the case” in developing countries for supporting standards – especially if this lowers barriers to local innovators sharing in health technology value chains.

This Background on global health technology standards has established how they can be beneficial and why people might want to create and use them. Next, the Discussion will explore what we can learn about standards from other industries.

## Discussion

We argue that other industries have much to teach us about developing global health technology standards. This is important, since such standards can create significant health benefits worldwide. If standards lead to lower-cost health technologies, then a given health budget can produce more health benefits. And if standards lead to more rapid innovation in global health technologies, then the set of health technology options will expand, which can likewise increase health benefits.

Examples discussed below include electricity, container shipping, CD standards, Universal Serial Bus (USB), and the Internet. In the supplementary text file, we explore two other examples in more depth: cell phones and semiconductors [see Additional file 1].

All these industries suggest how interface standards and interoperability can assist development of global health technologies. After the examples, we summarize lessons for global health technologies, and apply these lessons to the specific case of point of care diagnostics.

Before discussing lessons from other industries, we note the tremendous amount of work done over the last decade in health care technology standards for devices and data [33]. Examples include device-level standards (IEEE 11073, Bluetooth) and medical information exchange standards (HL7) [34]. Interoperability has been argued to be critical for exchanging health information [35], data sharing in research and healthcare [36], and the use of standards to support clinical research [37]. Many entities have arisen focused on health information technology standards – some part of established groups like the IEEE, others stand-alone organizations or consortia [38]. While our focus is on lessons from other industries (especially those where standards-based global implementation is at least a decade old, to better distill durable lessons) we feel that these health technology standards efforts hold lessons supportive of our general argument.

### Case studies from other industries

The brief standards development case studies in this section are drawn from industries in which standards have led to widespread adoption and innovation for at least a decade. The cases were chosen to illustrate the evolution of platforms that are used across an industry, to be well-documented, and to have lessons germane to global health technologies.

The cases (and the lessons which follow) are discussed from both *economic* and *technological* viewpoints, illustrating incentives and design principles respectively.

#### Shipping containers: efficiency in a box

From 1958 onward, US and other agencies worked to end the anarchy of different shipping container dimensions from different carriers – a variety which complicated logistics and intermodal transfer. The US Maritime Administration (MARAD) had significant market power due to both regulatory authority and provision of subsidies. The committees tasked with deciding on a standard had many different proposals, reflecting the interests of proposing parties (e.g. existing container stock, and preferences depending on where each party normally carried freight) [39].

A MARAD committee agreed on one set of sizes, while a defense-related task force decided on other sizes for military cargo. After a tangled and at times contentious process involving industry, government, and defense sectors over the next few years, US standards were achieved, followed by international standards through the ISO (International Organization for Standardization).

*Economic viewpoint:* the story illustrates how parties with buying power can influence standards – a common situation in global health technologies with large public or donor buyers. The standards reflected compromises, but were “good enough” for major parties and enabled long-term economic gain once accepted. For example, the cost of loading cargo onto a ship has over the years dropped by more than 80%, which made international trade significantly more cost-effective. This helped change global patterns of economic growth through trade [40].

*Technological viewpoint:* engineering tests played an important part in modifying the standard, such as when corner fittings had to be revised after failing stress tests simulating conditions in diverse parts of the world. Standardized shipping containers also enabled process innovations such as intermodal transport without unpacking. Unanticipated uses arose such as repurposing end-of-life containers for low-cost housing – a situation replaying itself in global health as mobile technologies are repurposed for health applications.

#### USB: plug and play

The concept of “platform leadership” has as a tenet the market leadership possibilities in creating a standard platform that others build on – a strategy followed by Cisco, Intel, Microsoft, and others [41]. Such platforms have sometimes been created as open Intellectual Property (IP) spaces where any innovator could make use of the new standard. An example is the Universal Serial Bus (USB) standard, for which Intel instigated the development process in 1994 with 6 other large companies including IBM and Microsoft – a small, closed group where each partner had significant assets and credibility with the broader market.

*Economic viewpoint:* getting the USB standard adopted required marketing and evangelism with diverse industry participants, including software and hardware developers and peripheral manufacturers. This marketing was assisted by uptake from key partners, e.g. Microsoft implementing USB in its operating system. Such uptake acts as a strong credibility signal – one which might be sought by parties advocating new global health technologies. The IP approach taken was to license USB royalty-free to other parties, as opposed to the less stringent RAND (“reasonable and non-discriminatory”) terms often required by other standards bodies.

*Technological viewpoint:* the humble USB plug represents a remarkable innovation – one that has reduced complexity and made life easier for everyone who uses a computer. Before the advent of USB, there were a plethora of connectors for peripherals. Now, many peripherals and devices can be interconnected worldwide using only one standardized plug – one that also supports daisy-chaining several devices in a row and recharging.

This high degree of interoperability required strong specifications and testing, including “plugfests” where manufacturers gathered to test devices with each other. Could health technologies be designed with similar interoperability and testing?

USB’s innovation platform is now being applied in novel ways. Examples include powering LED lamps and charging and communicating with medical devices.

#### Electricity: a platform for power

When electricity was first being set up as a power source in the US in the 1880’s, Edison’s DC system battled Westinghouse’s AC system. This was an early and infamous standards war [42]. Edison and his allies fought to convince the public that AC was less safe, even promoting an AC current electric chair for electrocution of criminals.

*Economic viewpoint:* ongoing innovation in AC and lower transmission costs helped lead AC to victory over the earlier DC technology. First-mover advantage can be overcome when users are not overly entrenched – a consideration when designing health solutions that outperform what already exists.

*Technological viewpoint:* once electricity systems and plugs were standardized, the electricity “platform” enabled progress over many decades [43]. Today, standards are being discussed for future smart energy grids which will enable multi-way sharing of information and electricity [44].

#### Color TV: competing standards

Black and white TV started broadcasting in the US in 1941, but as late as 1963 only 3% of US households had color TVs. RCA and CBS had competing technologies, both of which were costly. Technology adoption can be slow where price relative to performance is unappealing.

*Economic viewpoint:* the CBS technology was initially adopted in 1950 as the new color standard because the RCA technology had technical problems and a higher price point. It took another decade for color TV sales to become a large profit center, showing how new standards can diffuse slowly when existing solutions are “good enough” and incremental costs are high for buyers – a factor of particular relevance in the budget-constrained global health arena.

*Technological viewpoint:* RCA’s formation of an industry alliance and a standard that would be backward-compatible with black and white broadcasts helped reverse the standard in 1953. Similar to the AC-DC electricity battle, RCA’s technology and partnerships had overcome the CBS first-mover advantage [45].

#### Compact discs: domination and repurposing

Compact discs were a digital solution to music storage that grew to dominate the market. Compact disc standards were first released in 1980.

*Economic viewpoint:* Providing a precedent for health technology developers, Sony and Philips learned from history and avoided a standards war with compact disc technology [46]. Their effort was focused on convincing customers to invest in new players and discs. This enabled the market to take off relatively quickly, avoiding a repeat of the VHS-Betamax videotape standards war which harmed both consumers and manufacturers. Because avoiding standards wars can benefit consumers as well as companies, co-operation on standards can bypass antitrust laws against collusion.

*Technological viewpoint:* Sony and Philips worked together on R&D. Years later, compact disc technology was repurposed for data storage. Along with providing large amounts of data storage, this had the additional benefit of allowing computers to readily read and write the large “installed base” of music.

#### The internet: ubiquitous compatibility

The Internet is the ultimate example of the power of standards and interoperability.

*Economic viewpoint:* Devices which connect to the Internet are free to evolve as long as they support networking standards. This suggests that standards for “edge devices” should specify how devices interoperate, but not what additional functionalities the devices may have. In contrast, standards for telecommunications companies’ capital-intensive equipment incorporates its long-lived nature. When designing health technologies, aspects which are meant to endure or interconnect with other technologies may require tight specifications, but for as little functionality as possible.

The Internet could have evolved very differently. There were pressures in the 1990’s to create a “Web TV” which would only access content from a single provider and restrict users’ ability to create new content and services. Ultimately, the power of an open approach won out, illustrating the benefits when diverse providers can innovate with low barriers to entry. Debates today about future standards – e.g. platforms that are less open but promise more quality control and less malicious software – may yet change the current open nature of the Internet [47].

Positive network effects also played a critical role. Past some point, everyone wanted to connect to the Internet to reach all the other people who were already connected to the Internet. Those aiming to build new health technology platforms might consider how to stimulate interest and user communities at an early stage.

*Technological viewpoint:* A key concept in Internet standards is the idea of a “protocol stack”. Each level of the stack uses standards which handle some aspect of data communications or manipulation. Higher-level standards can be changed independently of lower-level standards.

For example, the details of how information is traveling – such as through a fiber-optic cable, a phone wire, or a wireless connection – are abstracted into the idea of a communications pipe at the base of the protocol stack. Functionalities like checking received data for errors and requesting retransmission of garbled information can then be defined independently of the particular physical means of transmission employed. This approach is a powerful one for standard-setters to implement where possible: define logically independent layers, each of which interacts with others only via specified interfaces.

A standards failure related to the Internet was the X.400 email specification that aimed to interconnect proprietary email systems. It failed for several reasons: premature and complex specifications, inadequate first implementations which may have deterred early adopters, and lack of incorporation of technical progress into the standard. The X.400 story illustrates why standard-setting needs to track technical progress. It also suggests the dangers in complex and all-embracing standards as compared to more modular and extensible ones [48].

### Lessons for global health technologies

This subsection suggests lessons for global health technologies, drawing from the case studies above (as well as others in the Additional file and the literature [49,50]). It applies many of the lessons to point of care diagnostics as a case example.

While reading the lessons, the reader may wish to keep in mind several barriers to standardization for global heath technologies such as diagnostics and medical devices. First, the landscape of health technology developers is fragmented, comprising developers which are small and large, for-profit and non-profit, and from higher-income and lower-income countries. This diversity and lack of dominant entities may impede standards efforts. Second, incentives of for-profit and non-profit entities may differ – for example, if for-profit companies foresee higher profits from locking users in to their product platform (even if it means users pay more and hence have less access to health technologies). Third, while quality standards have long been built into health products, interface and platform standards are relatively new to the field. Fourth, the diversity and ongoing evolution of global health technologies hinders standardization. Fifth, health needs and delivery systems differ across geographies — e..g. a point of care test for a remote rural location "under the tree" must be designed differently from a test for a health facility with some infrastructure.

#### Economic viewpoint

*Innovation potential:* Familiar platforms such as electricity and the Internet enabled huge innovation over decades. They also enabled long-term economic gain, and innovations in business models. A diverse range of content providers built on the platforms, spurred by low barriers to entry. For global health technologies where monetary considerations are important, thought should be given to incentives for platform innovators. Interest and user communities should be stimulated at an early stage.

*Open platforms:* In some cases – the Internet, telephones, TV – it is clear that, after an initial technology development stage, adopting a single standard can reduce complexity and cost and foster efficiencies. Suitable health technologies might have architectures that can be modularized and benefit from innovation by diverse parties. A standardized installed base lowers barriers to entry and allows innovators to create specialized parts of the modular technology without needing to create an entire technology platform.

Open platforms and standards could benefit diagnostics. An example is the open Medical Application Platform approach which seeks to provide device and health information interoperability, safety-critical functionality, and the ability to run clinical applications—all of which could offer advantages for safety and effectiveness in health care delivery (including point of care diagnostics) [51]. The widespread adoption of open platforms like this could potentially reduce the duplication of effort and investment inherent in creating a separate platform for each medical device.

*First-mover advantage:* First-mover advantage is important, especially in markets with strong network effects where the value of a choice increases as others make the same choice. However, as illustrated by several examples, first movers can be displaced by later comers with superior technology or business strategy – as long as users are not overly invested in the first mover’s technology. Having a backward-compatible standard helps. (An example from global health is the superseding of ordinary bednets by long-lasting insecticide treated nets. The superiority of the latter allowed them to overtake the former, while the similarity in form factor eased the transition [52]).

*Building support:* Building a customer base to support a new standard can be aided by seeking early adopters, offering compatibility with other market-leading standards, and investing to create an installed base of platform users. Bringing partners and customers on board can be assisted by recruiting prominent allies, evangelizing, and utilizing peer review and open trials to ensure a good standard.

*Ongoing commitment:* Creating health standards and standardized health technology platforms is not a one-time investment. If a standard or platform becomes ossified, imposes too much overhead, or suffers from security flaws due to its openness, a new player may introduce a proprietary alternative that provides a superior user experience. A useful standard evolves in tandem with technological and economic realities, and requires ongoing commitment. In the global health field, there may be tension between this ongoing commitment and the grant-driven nature of much health technology innovation. Even large foundations will have to credibly demonstrate their “staying power” if they wish others to adopt standards they propose.

*Significant benefit:* New standards can diffuse slowly when existing solutions are “good enough” and incremental costs are high. Conversely, if major players create an improved standard, it can spread quickly. This suggests an important role for large funders to kick-start health technologies. Even with such support, the standard should offer significant value increase at an affordable cost for manufacturers, buyers, and users in the field – and “cost” can include money, time, and other aspects.

*Strategy:* As standards develop in an industry, there are three types of outcome: collaborative adoption of a single standard, an oligopoly where multiple standards persist, and a standards war [53]. Many assets come into play in deciding each party’s strategy: a loyal or locked-in user base, IP rights, innovation and manufacturing capability, first-mover advantage, strength in complements, brand name and reputation, and pressure from buyers and funders. From a global health point of view, avoiding competing standards may speed the introduction of affordable life-saving technologies – but only if the standard adopted promotes innovation and is widely deployed.

*Rapid uptake:* New technologies which do not require a co-ordinated switchover have had rapid uptake, especially when immediate benefits are evident. This dynamic could aid the adoption of health technologies which are backward-compatible; which interact with other parts of the health system through well-defined interfaces; and which can be adopted in a decentralized way (for example, by different health providers).

An example is the development of a low-cost imaging solution which is a simple attachment to a cell phone [54]. The attachment is claimed to convert a cell phone to a microscope which can image both stained and unstained blood-smears, allowing diagnosis of clinical pathologies. If future iterations of such attachments become available cheaply with high quality and ease of use, they might rapidly become widely used in point of care settings around the world—not least because they can be adopted in a decentralized way with low incremental cost, rather than requiring entire health systems to switch over technologies.

*Reducing unnecessary costs:* Incompatibilities can arise from “good enough” decisions, yet persist for decades and require large costs to ultimately reconcile. When standards are adopted and fail, such as with the Betamax loss to VHS, those on the losing side eventually write off their sunk costs and move on. The aggregate losses of such sunk costs illustrate why a successful standard-setting process has economic and social benefits: avoiding unnecessary standards wars reduces unnecessary costs. For new global health technologies, it will be important to avoid unproductive standards wars, or cases where a standard platform is put forth and eventually fails. This in turn requires effective standard-setting processes.

#### Technological viewpoint

*Effective convergence:* An effective standard-setting process assists convergence to a single standard. This encourages all parties to incorporate the standard into their product designs. It also helps ensure that the chosen standard is well-designed from economic and technological points of view.

*Leapfrogging:* Some early standards have failed, such as early email and color TV technologies. More subtly, successful early standards can impede adoption of later standards. The French Minitel electronic network had tens of millions of users in the 1990’s, but its large installed base meant that later the French were relatively slow to adopt the Internet. Those procuring health technologies may at times have to decide whether to wait and adopt a later version of a standard, potentially “leapfrogging” a lesser-developed earlier version.

*Modularity:* The Internet’s “protocol stacks” illustrate a layered form of modularity on which medical devices might build. Modularity enables subparts to be innovated independently and incrementally, and it helps mitigate complexity. However, it has been found empirically in several R&D-intensive industries such as semiconductor design that there are limits to the modularity approach in practice. These limits will need to be clarified for global health technologies.

The open platform and modularity lessons suggest that complexity-reducing modular architectures may ease entry barriers for creators of new global health technologies. For example, a new sensing module based on novel biomarkers or novel surface properties could be integrated with an existing point-of-care diagnostic device, if the latter had a modular and interoperable architecture. Similarly, a series of simple sample preparation modules capable of processing and concentrating various specimen types (e.g. blood, sputum, saliva, stool), each with a common interface to a generic cartridge for analysis (e.g. Polymerase Chain Reaction (PCR) of sample DNA) would make the diagnostic platform more useful and prevent users in resource-limited settings from having to purchase multiple single-use diagnostic systems.

*Interoperable ecosystems:* One can move beyond a single device and consider an ecosystem of interoperable devices. A high degree of interoperability requires strong specifications and testing, as with USB “plugfests”. If achieved, it enables technologies to be used in ways that might not have been originally anticipated. Mobile health is a familiar global health example of this technology "repurposing" [55]. Standard-setting processes for a global health technology should include sufficient engineering acumen to implement strong specifications and testing.

An example of interoperability is the growing deployment of heterogeneous point–of-care diagnostics that interact with other healthcare infrastructure, to enable better monitoring, diagnosis, and research [56]. It should become increasingly possible for devices to interconnect and share information, without constraining the functionality that each device provides – a strategy that worked well with telecommunications systems and the Internet.

*Collaboration:* Developing standards as part of a larger consortium or pre-competitive collaboration can mitigate risks and avoid inappropriate standards. In the semiconductor industry, pre-competitive collaboration was supported through funding long-term initiatives, linking academia and industry, and motivating talented people to take part.

For diagnostics, a senior industry source has suggested realistic ways for firms to collaborate: “Companies could more widely share the details of their internal quality systems, enhance standards for reporting adverse events and corrective actions, standardize the reporting and handling of incidental findings, and publicize best and less-than-best practices” [57]. Motivations for such sharing could include regulatory requirements and pressure from buyers, as has already happened in standardizing aspects of analysis and reporting. (The same source indicates that other aspects that are competitive differentiators, such as assays and interpretation formats, would be harder targets for collaboration).

*Technology foresight:* Technology road-mapping and related foresight methods may enable pre-competitive collaboration for health technologies [58]. They can help in breaking down a technological challenge into sub-goals, and in envisioning scenarios for which global health technologies might be designed. Simulations and design methods can help in making user-centered trade-offs, and answering questions like: what are the key interfaces and components? Could point-of-care diagnostic devices use USB for charging and communication? What are the product profiles implied by a need for robustness and simplicity [59]?

Imagine design trade-offs for a standards-based point-of-care diagnostic. One party might be satisfied with a cheap and unattractive version, while another might pay a premium for a small, slick, and rugged version. The same components might be combined into larger versions, such as a stationary multi-sample unit able to process many samples simultaneously. There may be no single device and modular attachment (e.g. a microfluidic chip) which meets everyone’s needs, leading to trade-offs and hard decisions when standardizing. In the course of complex discussions of technology, there is a need for smart systems architecture thinking – “option mapping” that clearly lays out design considerations, and facilitates effective decision-making.

### How to develop standards

Thus far we have argued in favor of developing standards for global health technologies by learning lessons from standards in other industries. But a skeptical reader might object that the *process* of creating global health technology standards is impractical, involving too many challenges and unknowns.

In the remainder of the Discussion we therefore discuss intellectual property and regulatory considerations and standards-based global health business models, and end with a checklist of considerations for health standards developers.

We note that the experiences of some standard-setting organizations clarify how one might develop health standards. For example, CLSI (the Clinical and Laboratory Standards Institute) develops “…globally applicable voluntary consensus documents for health care testing” [60], such as consensus guidelines for analysis and presentation of antimicrobial resistance trends [61]. It has been selected by WHO as a collaborating center for clinical laboratory standards, and it has detailed procedures for developing consensus standards and guidelines [62]. Two timelines used are 15 and 25 month tracks, with the former for non-controversial projects with narrow scope. The supplementary text file further explores standards development organizations and processes [see Additional file 1].

#### Intellectual property and regulatory considerations

Standard-setting activities frequently run into IP considerations [63]. For example, a standard may be set and specified, with parties only afterward learning that one party holds a crucial patent [64]. Standard-setting initiatives should discuss IP and confidentiality provisions with key parties at the start of the process, and learn from provisions adopted in previous standards efforts.

While patents are often viewed as promoting innovation, questions have been raised surrounding IP, innovation, and international development [65,66]. For example, when should patent protection be relaxed for humanitarian reasons, and what barriers to follow-on innovation might the IP system itself create? Innovation rests on a public domain of ideas [67]. Patent pools (consortiums which cross-license patents related to a particular technology) are beginning to be used to stimulate research in neglected diseases, allowing both access to technologies and viable business models [68]. More strategies will be required to ensure that the IP landscape for health technologies rewards innovators while accommodating humanitarian and economic goals in developing countries [69].

It can be hard to ensure that standards for each component of a standards-based solution translate into a high-quality overall solution. For example, the automotive industry has experienced systems integration challenges when auto parts from suppliers interact in unforeseen ways or have subtle flaws that are difficult to test for [70].

This challenge might be addressed through both a *formal testing process*, and *open test suites* against which manufacturers could verify compliance. Formal testing can be required by regulatory authorities, or by buyers who seek higher quality than the regulatory minimum. Open test suites could be an opportunity for pre-competitive collaboration, especially if health technologies certified against these open test suites are favored by regulatory authorities or large buyers.

Aside from regulatory controls, quality can be promoted via voluntary codes, though some such codes may set a low quality bar [71]. Quality can also be promoted via reputation systems that allow users to rate the quality of goods and services, though such systems are subject to manipulation and inaccuracy [72]. Voluntary codes and reputation systems may supplement formal regulation if lower entry barriers draw diverse developers, as might happen if a standards-based point-of-care device allows open development of downloadable diagnostic algorithms.

This “opening up” of a medical device platform raises many regulatory issues. It has been argued that open platforms are more vulnerable to attacks and infiltration, and counter-argued that open platforms invite greater scrutiny and more rapid patching of weaknesses. The complexity of computer and device security threats suggests that there are subtle arguments on both sides, and that the issue will be increasingly important in developing health technology standards [73].

#### Standards-based global health business models

What do the standards lessons and arguments above imply about business models for global health technologies?

End users generally have low ability to pay for global health technologies, which generally have small markets. In lower-income countries, R&D funding for these technologies comes mainly from public and donor sectors, with a small but increasing private-sector contribution. In higher-income countries, funding comes from governments, philanthropies, and technology developers [74]. As emerging economies grow, their contribution to health technology funding will increase.

Standards-based platforms may reduce the cost of entry for health technology developers. They might, for example, specialize in consumables, modules, or software for a diagnostics platform. Ultimately, lower-cost platforms and components can increase access for populations which could not afford the previous generation of more expensive technologies.

Uptake of a global health technology standard may be affected by its applicability to a higher-income market. To take an example, low-cost open-standards diagnostics may not displace higher-end diagnostics in higher-income markets in the short term, as they may not match performance and functionality. However, new modular diagnostics platforms may eventually become a disruptive innovation globally.

Requirements for effective diagnostics in the developing world have been divided into three cases with no, minimal, and moderate infrastructure (with point-of-care diagnostics particularly needed in the first two cases) [75]. To enable better-informed business models, could a *shared use case library* be developed?

This might comprise both explicit requirements like having to function with no or intermittent electricity, and tacit knowledge like interviews from clinicians sharing their practical requirements and socio-cultural concerns. Might this help diagnostics developers to build more effective devices and businesses based on past experience?

#### A checklist of considerations for health standards developers

Developing a useful standard which achieves broad acceptance poses many difficulties: mustering support, resolving interoperability issues, working with installed technology, and more. Repeating the mistakes of past standards development efforts should not be one of these difficulties.

We therefore propose a checklist of considerations for health standards developers. These questions were developed iteratively by the authors. We drew from the literature and arguments summarized in this article, from our personal standards experiences in health, semiconductor, and other industries, and from the checklist method that has proven its value in health and elsewhere [76]. We also drew from the rules of thumb for creating standards which one of the authors has developed into a book [77].

**This checklist does not constitute legal advice.** These considerations are suggestions only.

*Before you start:*

– Is there a 5-year organizational and financial commitment from you as the lead party?

– Is there a strong need for a standard? Will the standard lead to less expensive and more effective health technologies for end users, and hence more lives saved?

– Have customers/suppliers been identified who will implement the standard in their products?

– Is the underlying technology sufficiently mature and field-tested to allow standardization?

– Is someone on your team familiar with the history and practice of technical standards?

– Has a similar standards effort been tried before? Are there lessons to learn to avoid pitfalls?

– Are there competitive forces that should be considered?

– Can you join an existing consortium or other collaborative effort?

– Will accreditation by a recognized, international body be required?

– Would technology foresight methods help clarify challenges, opportunities, and use cases?

– Is the scope of the effort reasonable so as to ensure its completion in a timely manner?

*As you develop the standard:*

– Have you clarified which aspects of the health technology are candidates to be standardized, including technical, business, and regulatory aspects?

– Are there proven technologies that can be used as a basis for the standard?

– Are leading technical and business perspectives represented in meetings?

– Are meetings and standard-setting processes productive, effectively run, and enjoyable?

– Have you clarified Intellectual Property and confidentiality provisions, and regulatory issues?

– Have best practices been learned from previous standards efforts?

– Are early adopters, user communities, and general interest being stimulated at an early stage?

– Does the standard promote backward-compatibility, extensibility, interoperability, modularity, maintainability, device-independency, internationality, and simplicity?

– Do you have public buy-in from key funders and decision-makers who can accept and promote the standard?

*Before finalizing the standard:*

– Has constructive criticism been sought from key stakeholders for the draft standard?

– Is there buy-in from enough parties to make widespread adoption of the final standard likely?

– Have technical, business, social, and cultural factors been explored, and risks mitigated?

– Has the standard been tested in the lab, in the field, and in peer review and credible trials?

– Have regulatory and safety issues been addressed?

– Does the draft standard offer significant value increase at an affordable cost for manufacturers, purchasers, and users?

*After the standard is finalized:*

– Are you (and any key partners) stewarding the adoption and development of the standard?

– Is field experience gained from implementing the standard being shared among technology developers and researchers?

– Are there clear incentives and business models that support the standard?

– Are there few entry barriers to using the standard, especially for new implementers?

– Is there a plan to promote, market, and/or advertise the standard?

– Is there a training program and/or users’ manual available for new adopters?

– Has the standard enabled more affordable and effective health technologies?

– Are partnerships with developing countries being utilized, for both development and delivery of novel health technologies and standards?

– Have you built in a periodic review process for the standard, to maintain its relevance as technology, markets, and health conditions evolve?

We do not claim that this checklist is perfect. (Indeed, we invite readers to improve it.) But we do believe that this type of checklist can be useful, and that lessons from other industries and past standards development experiences can help to develop global health standards in the future.

### Summary

This article has shown how learning lessons from other industries can help us develop standards for global health technologies. We have explained why standards are needed in global health, given a brief primer on standards, and examined incentives to create and use standards. We then explored lessons from other industries, considered the standards development process and standards-based business models, and gave a suggested checklist of considerations for health standards development leaders. Point–of-care diagnostics were used as an illustrative example throughout the article.

The development of mutually incompatible solutions by different organizations can slow the pace of innovation, lock health systems into single providers and approaches, and keep barriers to entry high. These problems can be mitigated by effective standards. In diagnostics and other global health technologies, standards help enable innovation and affordability.

Experience from other industries suggests that global health technologies conforming to well-chosen standards can enable new entrants and lower price points, link islands of innovation, and create vibrant solution ecosystems. Realizing this potential requires support from diverse stakeholders.

R&D funders and global health organizations can provide leadership and funds. Policy-makers can support standards-based approaches. Technology developers can consider interoperable approaches, and look for opportunities to interconnect with existing technology components. Those in charge of procurement can pressure suppliers to provide interoperable and modular solutions. Suppliers can in turn design technologies to be interoperable, engage in pre-competitive collaboration, and try more open business models where feasible.

We believe that point-of-care diagnostics can be one of the first global health technologies that benefits from this approach at global scale – especially if lessons are learned from other industries. By 2025, could a diagnostics revolution have as much impact on worldwide healthcare as the cell phone did on worldwide communications? Could other global health technologies follow suit? The health needs of the 21st century demand nothing less.

## Competing interests

The authors declare that they have no financial competing interests. RL works for the non-profit organization Grand Challenges Canada, which is contributing funding to explore standardized diagnostics platforms for global health. KB is the 2013–2014 President of the non-profit IEEE Standards Association, and author of *The Ten Commandments for Effective Standards: Practical Insights for Creating Technical Standards*.

## Authors’ contributions

HM designed and researched the article, and wrote the first draft. HM, RL, and KB contributed to the content of the article, analyzed and refined the argument, and participated in manuscript development. All authors read and approved the final manuscript.

## Supplementary Material

Additional file 1 Insights from two additional cases of standards, and discussion of the standards development process. Click here for file
