# Supplementary material for: Developing global health technology standards: what can other industries teach us?
Source: Global Health. 2013 Oct 17;9:49. doi: 10.1186/1744-8603-9-49 (PMC3853011; doi:10.1186/1744-8603-9-49)
Supplement: Additional file 1 — Insights from two additional cases of standards, and discussion of the standards development process. [file 1744-8603-9-49-S1.pdf]

Additional file 1 for article "Developing global health technology standards: what can other industries teach us?"

This supplemental file has two parts. In "Lessons from standards in other industries", we discuss insights from two additional cases of standards. Then, in "Developing global health technology standards", we discuss the standards development process.

Key points of "Lessons from standards in other industries"

- Semiconductor design suggests that modularization, reuse, and pre-competitive collaboration help handle complexity and cost. However, systems integration (combining modules to make a well-functioning whole) can be challenging.
- Telecommunications demonstrates the potential value of a globe-spanning network of interoperating components. This happened several times worldwide, including the telegraph, telephone, cell phone, and Internet.

Key points of "Developing global health technology standards"

- In setting standards, there are decades of knowledge and failures to learn from. Key considerations include costs, communications, stakeholder benefits analysis and engagement, and forming a core development group.
- Over decades, standard-setting organizations have refined their approaches for bringing diverse parties together to create standards. Most of the work of the ISO (International Standards Organization) is done in decentralized technical committees. The IETF (Internet Engineering Task Force) aims for "rough consensus and running code", rather than lengthy processes that yield theoretical standards. Principles of the IEEE-SA (IEEE Standards Association) include due process, openness, consensus, balance, and right of appeal.
- Innovation from the global South can help implement and deliver standards-based global health technologies. A more modular and interoperable approach to global health technology can open niches for Southern partners to innovate lower-cost and customized components. Southern innovators are closer to the front lines of health technology application—they have local cultural and regulatory connections, R&D resources, cost efficiencies, and the potential to customize and co-brand health solutions for local markets.

Lessons from standards in other industries

In this supplemental section, we discuss insights from two additional cases of standards in other industries. These cases each illustrate a feature relevant to global health: modular platforms, and large-scale interoperability.

Cases are discussed from two perspectives: *economic* and *technological*. Both these perspectives are essential to understanding when and how to implement standards.

"Plug and play": modular platforms

Building on examples drawn from both high-tech and low-tech industries, this section focuses on a specific type of standard that we might call "plug and play platforms". To understand such platforms, one must first understand the concept of modularity.

A *modular architecture* is one where components are designed to be as independent as possible and interact only through well-defined interface methods. These interface methods should themselves be simple. As much as possible, they should depend only on the functionality provided by the module, and not on its specific implementation details. As Sanchez says, "Modular architectures are essentially a tool for achieving a range of potential benefits – greater product variety, faster technological upgrading, greater speed in bringing new products to market, and reducing product realization costs." [1]

Economic viewpoint: The semiconductor design and manufacturing industry has sales of hundreds of billions of dollars per year of microprocessors, memory chips, and other components. It faces challenges including the rising cost of design and manufacturing, intense competition, potential limits to continued performance increase, and high risks of innovation [2]. Moore's Law – that the number of transistors on a single chip doubles about every two years – has held for several decades, but poses formidable problems to maintain [3].

The rising cost of manufacturing and design has driven modularization and reuse, which in turn was enabled by and facilitated standards [4]. "System on a chip" design requires many different components to function independently while interoperating on a single chip. While a system on a chip can have benefits in power, size, speed, reliability, and production cost, the costs of design are higher.

One reason is that it has become more difficult to design a highly complex new system on a chip than it is to manufacture its more than 1 billion transistors. (It has been suggested that the complexity of technologies like semiconductors can be advantageous to incumbents – limiting entry opportunities for developing countries and start-ups, which must invest heavily to gain specialized expertise and resources [5].)

Another reason that design costs are higher is the greater constraints on power use and performance – chips in modern electronic products must run faster while consuming less power. All this is driving reuse of "design cores" (chip subunits), both within and between companies.

Pre-competitive collaboration worked in this capital-intensive and rapidly-evolving industry. Indeed, the International Technology Roadmap for Semiconductors regularly brings together hundreds of skilled parties to set R&D directions and challenges for the industry [6]. In the semiconductor industry, this collaboration was driven by the challenges identified above, including high capital costs, risks, and complexity. Health technologies with similar attributes may particularly benefit from pre-competitive collaboration.

Technological viewpoint: Interoperability promotes innovation. Modularity enables interoperability, by allowing subparts to be innovated independently and incrementally. Components can be developed separately by different parties, who can tackle one well-defined technical challenge without needing to innovate across an entire system.

Traditionally there has been a standardization–adaptation trade-off by multinational companies, which balance using the same design globally against adapting designs to local preferences. Modularity and the related feature of programmability allow functional versatility without overly increasing cost [7]. Indeed, modularity helps mitigate complexity.

A plug and play platform, then, could be thought of as providing basic intrinsic functionality, along with standard interfaces to connect other modular components for additional functionality. The electricity system might be thought of as one such "platform" – when the standardized system and receptacles were implemented many decades ago, there was little inkling of today's plethora of plug-in electronic and computational devices. Another familiar example is the smartphone application marketplaces that exist for iPhone, Android, and Blackberry. Standardized functionality from the smartphone – combined with standard interfaces by which applications can use the smartphone's functionality – have enabled an explosion of innovative applications in a short space of time, including in the burgeoning field of mobile health [8].

In semiconductor chip design as well as other fields, there are in practice limits to the modularity approach [9]. Systems integration (i.e. the ability to combine modules into a well-functioning whole) hampers creating effective plug and play architectures. This has empirically been found to be a challenge in semiconductor manufacturing, as well as in the automotive and airplane industries [10].

A point to watch in establishing a platform for a new global health technology will be the degree to which modularity is possible in practice. This will likely require both technical expertise and experimentation to establish.

Telecommunications and cell phones: large-scale interoperability

Economic viewpoint: Telecommunications hardware and protocols demonstrate the potential of a globe-spanning network of interoperating components. This happened several times worldwide, including the telegraph [11], telephone, cell phones, and the Internet [12].

Regulated landline monopolies in many advanced economies initially controlled both the systems and the attached devices. In 1978 in the US, interface standards were specified which let consumers purchase phones from any approved manufacturer. The US and several other large countries then broke up their telephone monopolies in the 1980's.

Subsequently, cell phone transmission standards (i.e. standards for wireless communication between cell phones and base stations) evolved differently in America and Europe. The process by which these standards were created itself evolved over time [13]. The first generation of cell phone transmission standards was implemented by regulatory agencies in the US and Scandinavia, via an open process and co-operation from manufacturers. These countries saw increased cell phone uptake, which convinced other European countries to adopt a more open and manufacturer-centric standard setting process for the next generation of standards. The pan-European GSM standard followed, driven also by European integration and competition with the US and Japan.

America allowed a market-based approach for its second generation of standards, where operators could choose which standard to support (e.g. TDMA, CDMA, GSM). Multiple standards may have been perceived to encourage more price and technological competition. Partly due to its early pan-European market share, GSM enjoyed greater global uptake in a self-reinforcing pattern as the developing world rapidly adopted cell phone technology in the 2000's.

Positive *network effects* promote market share. (The classic example of network effects is the fax machine: one fax machine is useless, but its value increases the more fax machines there are.)

Technological viewpoint: A mitigating factor preventing the American approach from suffering unduly from the multiplicity of standards was the ability to interconnect. No matter which standard one's local company supported, one could call any other wireless or landline number. The details of converting between standards were handled behind the scenes [14].

Cell phones illustrate another aspect of standards: where multiple levels of technology interoperate, at which level(s) do the standards apply? For example, base stations for cell phone networks are expensive. Changing "backbone equipment" across a network is a major financial and logistical undertaking. In contrast, handsets can evolve rapidly as long as they support the appropriate standards for wireless networking, and handset innovation has been rapid and sustained.

Coming to the present, we see the spread of open mobile standards such as Android. One risk of this approach is loss of control over quality of the user experience.

Developing global health technology standards

This supplemental section draws on previous experience in health and other industries to show how global health technology standards can realistically be created. It also discusses challenges in the standards development process.

Standard-setting processes can help competing parties find points of mutually-beneficial collaboration. They have even inspired a distributed-intelligence approach to global problem-solving [15].

Process considerations

Standards should have a strong economic, safety, or public benefit driver. The reason why a standard is needed should be understood by all parties. It should also be understood which parties involved will receive value, and how. In setting standards, there are decades of knowledge and failures to learn from, often embedded within veterans of standards processes [16].

- *Stakeholder Engagement:* To succeed, a health standard should satisfy at least four groups of stakeholders: users, developers, regulators, and funders. While open processes may be slower and potentially less focused, they can support transparency, broad feedback, and a perception of legitimacy.

- *Core group*: While many parties may be involved, it may be a core group with time, energy, and resources that sees a standard through. If such a core group never forms, and participants drop off rapidly, this may be a sign that the standards process is not worth doing.

- *Communication:* When parties are in multiple locations, collaboration technologies can help. Examples include email, threaded and archived discussion forums which act as a "group

memory", Wikis, teleconferences, videoconferences, and platforms such as Kavi which are designed to support standards processes.

- *Costs:* "Open standards" are not free. Travel and venue rental can be significant. Another cost is time: each engaged participant from an industrial, academic, or non-profit concern has to put energy into the process. If one includes the cost of participant time, creating and promulgating a complex standard can absorb millions of dollars.

Sometimes the mere existence of a standard is more important than which standard is chosen, e.g. which side of the road we drive on [17]. These can be thought of as *co-ordination challenges*, in which parties are not strongly vested in mutually incompatible options.

The more difficult type of standard to create entails *co-operation challenges*. For these challenges, parties may stand to lose customer base, incur significant switching costs, or otherwise lose competitive advantage. In this case, co-operating to reach a standard can require leadership, experience, connections, economic heft, and other strengths [18].

While standards for health technologies often require difficult co-operation challenges to be overcome, opportunities can be sought to overcome co-ordination challenges instead. Safety standards with global applicability can be created. Quality standards relevant to procurement decisions can be developed (e.g. for long-lasting bednets by WHO). Common standards for an early-stage technology can be agreed upon before any party has large sunk costs to recoup.

Governance of standard-setting

Those wishing to create or support standards can benefit from the experiences of effective standard-setting organizations (SSOs). SSOs have shown their value over the decades [19]. Over time, SSOs have refined approaches for bringing diverse parties together to create standards. We give three examples below to complement the example of CLSI given in the main text.

ISO (the International Standards Organization) has national standard-setting bodies as members. These bodies may themselves contain thousands of members, including government bodies, universities, companies, associations, and sometimes individuals [20]. Most of ISO's work is done in decentralized technical committees. A committee works to reach consensus and may require a two-thirds vote and other conditions to accept a standard.

The *IETF* (Internet Engineering Task Force) was founded in 1986. It aims for "rough consensus and running code", rather than lengthy processes that yield theoretical standards: "The process is believed to be as short and simple as possible without sacrificing technical excellence, thorough testing before adoption of a standard, or openness and fairness." [21] In contrast with many other standards bodies, much of the work of the IETF takes place in open mailing lists and Working Group meetings [22].

When the IETF identifies a new area for standards, parties rapidly start implementing and gaining experience. The process is an iterative funnel, from many proposed standards to few full standards. An example of the latter is the basic TCP / IP protocols on which the entire Internet runs – such ubiquitous standards have huge switching costs. There is much more room for experimentation and change at earlier stages.

The *IEEE* (the Institute of Electrical and Electronics Engineers) has roughly 400 000 members worldwide [23]. Standards are developed under governance of the IEEE Standards Association (IEEE-SA). Principles of IEEE-SA's standards setting process are due process, openness, consensus, balance, and right of appeal [24].

Once an IEEE draft standard has evolved to a stable state, it is voted on: "A standard will pass if at least 75 percent of all ballots from a balloting group are returned and if 75 percent of these bear a "yes" vote. If ballot returns of 30 percent are abstentions, the ballot fails." [25] Ballots can come with comments, each one of which must be responded to. Part of the art of reaching consensus is to consider which comments attached to negative votes might be addressed by changing the draft standard appropriately, thereby converting the negative vote to a positive vote.

Even from the brief descriptions above, one can see that different SSOs use different ways of structuring the stages of the standards development process. In practice, if a formal standards development process is used, then the stages as described by that standards development process or organization may be most relevant. See for example the referenced standards processes for any of the SSOs above, or the four informal stages we give in Table 1 of the main article.

While many standards groups require participating patent holders to license required technology under RAND ("reasonable and non-discriminatory") terms, this may be hard to adjudicate – e.g. what exactly does "reasonable" imply? Alternatives have been proposed such as patent pools or a time limit on patent assertions [26]. Risk-mitigating strategies of standard-setting organizations include mandatory disclosure of conflicts of interest and existing IP, and proactive compliance with antitrust laws [27][28].

Patent issues may be more of a risk in health technologies which combine innovations from different fields, such as a point-of-care diagnostic that incorporates cell phone, computational, and sample handling elements. "Regulatory stacking" may occur for such combination technologies, with multiple agencies each concerned with a technology-specific regulatory aspect of the product [29]. Health technologies meant for low-resource environments may also face regulatory barriers across diverse jurisdictions.

We end this subsection with a note on *de facto standards*, i.e. standards which have not received formal approval but nevertheless gain widespread adoption. An organization which has successfully created such a standard may continue this approach until the broader market shifts – for example, when customers start threatening to shift their business in search of more interoperability or choice. Global health funders might consider costs from a lack of interoperability when deciding whether to support particular vendors.

If de facto standards are widely available in a non-discriminatory and inexpensive manner, and encourage widespread innovation, then such standards can make an organization's technology available rapidly. This can work in tandem with formal standard-setting approaches. An organization may begin with a de facto (or "open proprietary") approach for speed and control. It might then submit the de facto standard to a more formal standard-setting process, for a collective "seal of approval" and fine-tuning from widespread peer review.

Utilizing Southern innovation to implement standards-based technologies

There is increasing capability for health technology R&D within many developing countries, including China, India, Brazil, and several African nations [30]. We refer to such innovation as “Southern innovation” – i.e. innovation from the global South. We argue that such innovation can help implement and deliver standards-based global health technologies.

Modular and interoperable technology can ease the partnering process. International partners can co-operate on a composite technological device without requiring full sharing of proprietary knowledge of each component. For example, a novel sample analysis module for a handheld diagnostic could interoperate with a platform created by another manufacturer. This may promote devices being repairable, adaptable, and recyclable with local facilities and expertise [31].

Technology innovators from the South might fruitfully partner with organizations from higher-income countries. Southern innovators are closer to the front lines of health technology application. They have local cultural and regulatory connections, R&D resources, cost efficiencies, and the potential to customize and co-brand health solutions for local markets. Partners from higher-income countries can offer experience, R&D expertise, financial resources, and standards development skills.

Another way of utilizing Southern innovation is to harness large-scale health manufacturing and delivery systems. One example is the Indian Immunologicals 3000-clinic system for rabies and other vaccines. Indian Immunologicals manufactures vaccines, and provides them along with training and quality control to doctors, who in turn contribute their existing facilities, expertise, and community networks [32]. Another example is the Aravind Eye Hospital’s low-cost eye surgery operation. Aravind manufactures its own lenses and uses process optimization and cross-subsidies to provide a large volume of high-quality surgeries [33].

Might large-scale health delivery systems be natural partners for development of standardized health technology platforms? They have proven ability to innovate and implement at scale. They might provide platform and device developers with large-scale field trial locations, multi-site metrics, and deployment partners. They might also manufacture standards-compliant components that are core to their health delivery services – analogous to what both Indian Immunologicals and Aravind have done.

References

[1] Sanchez R: **Using modularity to manage the interactions of technical and industrial design.** *Academic Review* 2002, **2**:8-19.

[2] Brown C, Linden G: *Chips and change: how crisis reshapes the semiconductor industry.* Cambridge, MA: MIT Press; 2009.

[3] Bohr M: **Moore's Law in the innovation era.** *Proc. SPIE* 2011, **7974**:797402-797408.

[4] Funk JL: **Systems, components and modular design: the case of the US semiconductor industry.** *International Journal of Technology Management* 2008, **42**(4):387-413.

[5] Brown C, Linden G: *Chips and change: how crisis reshapes the semiconductor industry.* Cambridge, MA: MIT Press; 2009.

[6] **International Technology Roadmap for Semiconductors** [http://www.itrs.net/]

[7] Lehrer M, Behnam M: **Modularity vs programmability in design of international products: Beyond the standardization–adaptation tradeoff?** *European Management Journal* 2009, **27**:,281-292.
[8] Vital Wave Consulting. *mHealth for Development: The Opportunity of Mobile Technology for Healthcare in the Developing World*. Washington, D.C. and Berkshire, UK: UN Foundation-Vodafone Foundation Partnership; 2009.
[9] Ernst D: **Limits to Modularity: Reflections on Recent Developments in Chip Design.** *Industry & Innovation* 2005, **12**(3):303-335.
[10] Prencipe A, Davies A, Hobday M (Eds): *The business of systems integration.* Oxford, U.K.: Oxford University Press; 2003.
[11] Standage T: *The Victorian Internet: The Remarkable Story of the Telegraph and the Nineteenth Century's On-line Pioneers.* New York, N.Y.: Berkley Books; 1999.
[12] Rowland W: *Spirit of the Web: The Age of Information from Telegraph to Internet.* Toronto, Canada: Thomas Allen; 2006.
[13] Funk JL: **The co-evolution of technology and methods of standard setting: the case of the mobile phone industry.** *J Evol Econ* 2009, **19**:73-93.
[14] Gandal N, Salant D, Waverman L: **Standards in wireless telephone networks.** *Telecommunications Policy* 2003, **27**:325-332.
[15] Rischard JF: **High noon: twenty global problems, twenty years to solve them.** New York: Basic Books; 2002.
[16] Bartleson K: *The Ten Commandments for Effective Standards: Practical Insights for Creating Technical Standards.* Mountain View, CA: Synopsys Press; 2010.
[17] Shapiro C, Varian HR: *Information rules: a strategic guide to the network economy*. Boston, Mass.: Harvard Business School Press; 1999.
[18] Calvert R: **Leadership and Its Basis in Problems of Social Coordination.** *International Political Science Review* 1992, **13**(1):7-24.
[19] Rysman M, Simcoe T: **Patents and the Performance of Voluntary Standard Setting Organizations.** *Management Science* 2008, 54(11):1920-1934.
[20] Murphy CN, Yates J: *The International Organization for Standardization (ISO): global governance through voluntary consensus.* London, U.K.: Routledge; 2009.
[21] **The IETF Standards Process** [http://www.ietf.org/about/standards-process.html]
[22] **The Tao of IETF** [http://www.ietf.org/tao.html]
[23] **History of IEEE [**http://www.ieee.org/about/ieee_history.html]
[24] **IEEE Standards Association - Developing Standards** [http://standards.ieee.org/develop/index.html]
[25] **IEEE Standards Association - The Balloting Process** [http://standards.ieee.org/develop/balloting.html]
[26] Rysman M, Simcoe T: *A NAASTy alternative to RAND pricing commitments.* Toronto, Canada: Working Paper, Rotman School of Management, University of Toronto; 2007.
[27] Balto, DA: **Standard Setting in a Network Economy** [http://www.ftc.gov/speeches/other/standardsetting.shtm]
[28] Clinical and Laboratory Standards Institute: *Clinical and Laboratory Standards Institute Administrative Procedures*. Wayne, PA; 2011.
[29] Saner M, Daar AS: **Streamlined Regulation of Converging Technologies.** *Optimum Online* 2011, **41**(3).
[30] Al-Bader S, Masum H, Simiyu K, Daar AS, Singer PA: **Science-based health innovation in sub-Saharan Africa.** *BMC Int Health Hum Rights* 2010 , **10**(Suppl 1):S1.
[31] **The Maker's Bill of Rights** [http://makezine.com/04/ownyourown/]
[32] Masum H, Batavia H, Bhogal N, Le K, Singer PA: **Franchising Rabies Vaccine Delivery: The Case of Indian Immunologicals.** *PLoS Neglected Tropical Diseases* 2011, **5**(4):e946.
[33] Rangan VK, Thulasiraj RD: **Making Sight Affordable (Innovations Case Narrative: The Aravind Eye Care System).** *Innovations: Technology, Governance, Globalization* 2007, **2**(4):35-49.
